# Perceptions of societal ageism and declines in subjective memory during the COVID‐19 pandemic: Longitudinal evidence from US adults aged ≥55 years

**DOI:** 10.1111/josi.12544

**Published:** 2022-08-07

**Authors:** Ella Cohn‐Schwartz, Jessica M. Finlay, Lindsay C. Kobayashi

**Affiliations:** ^1^ Epidemiology, Biostatistics, and Community Health Sciences Faculty of Health Sciences Ben‐Gurion University Beer‐Sheva Israel; ^2^ Center for Social Epidemiology and Population Health Department of Epidemiology University of Michigan School of Public Health Ann Arbor Michigan USA; ^3^ Social Environment and Health Program Survey Research Center University of Michigan Institute for Social Research Ann Arbor Michigan USA

## Abstract

The cognitive health of older adults since the COVID‐19 pandemic onset is unclear, as is the potential impact of pandemic‐associated societal ageism on perceived cognition. We investigated associations between perceptions of societal ageism and changes in subjective memory over a 10‐month period during the COVID‐19 pandemic. We collected longitudinal data from monthly online questionnaires in the nationwide COVID‐19 Coping Study of US adults aged ≥55 from April 2020 to January 2021 (N = 4444). We analyzed the data using multivariable longitudinal multilevel models. We identified an overall decline in subjective memory, especially in the initial months of the pandemic. Adults who perceived that societal respect for older adults decreased during the pandemic experienced more rapid declines in their subjective memory. These findings suggest that aging adults perceived a decline in their memory, especially during the initial months of the COVID‐19 pandemic. Societal interventions to combat ageism may help improve subjective memory and could decrease risk for cognitive decline among middle‐aged and older adults.

## INTRODUCTION

During to the COVID‐19 pandemic, ageism became highly prominent in the global discourse. People of older ages are at higher risks of serious illness, hospitalization, and death due to COVID‐19 (Wu et al., 2020). Consequently, discourse in the media and public health guidelines have portrayed “the elderly” as a homogenous and highly vulnerable group which requires shielding (Ayalon et al., 2021; McDarby et al., 2022), while neglecting the role of underlying health conditions and structural determinants of risk such as race, poverty and access to resources (Millett et al., 2020; Montero‐Odasso et al., 2020). Especially during the early pandemic (spring and summer of 2020), newspapers around the world were fraught with ageist content, ranging from naming the virus “Boomer Remover” to suggesting that older adults should sacrifice themselves for the younger generations and the economy (Ayalon et al., 2021). Accordingly, public discourse evaluation of tweets revealed the popularity of ageist tweets in the early wake of the pandemic (Jimenez‐Sotomayor et al., 2020; Xiang et al., 2021). Increased societal ageism was also reflected in perceptions of older adults as a “burden” due to healthcare systems becoming too overwhelmed with COVID‐19 cases to provide adequate care to all, especially before vaccine availability in the United States beginning December 14, 2020 (U.S. Department of Health & Human Services, 2022). The Centers for Disease Control (CDC) weekly reports tracking the COVID‐19 pandemic drew early attention to increased hospitalization rates among older adults. For example, the CDC (2020) report corresponding with this study's start of data collection in April 2020 reported that the overall cumulative hospitalization rate was 4.6 per 100,000, with the highest rates in persons 65 years and older (13.8 per 100,000) and those 50–64 years (7.4 per 100,000). By the end of 2020, the hospitalization rate among persons aged 65 and older was 66.9 per 100,000 (compared to 8.3 per 100,000 among US adults aged 18–49, and 25.7 among persons aged 50–64; CDC, 2022). Younger‐age care prioritization was evident in some healthcare policies and practices during the pandemic (Cesari & Proietti, 2020). For example, some countries, such as Italy and Sweden, have set age limits for providing intensive care treatment or ventilator machines for COVID‐19 patients in hospital settings, de‐prioritizing older adults (Ferraresi, 2020; Svensson, 2020; Truog et al., 2020).

These trends were reflected by older adults themselves reporting increased societal ageism during the COVID‐19 pandemic. For instance, older adults in Israel perceived ageism to be prevalent in society during the pandemic, especially the notion that older people are particularly vulnerable (Cohn‐Schwartz & Ayalon, 2021). Societal ageism was also seen in newspaper reporting from the United States during the pandemic, which contained ageist messages such as portrayals of older adults as “vulnerable” (Jen et al., 2021). A study by Kornadt et al. (2021), conducted among adults in Luxembourg, found ageism to be more prevalent in the media and healthcare settings, more so than in the personal social networks of adults. Furthermore, adults in that study who perceived societal ageism to be higher during the pandemic also reported lower life satisfaction and worse subjective health. However, that study did not look at the associations of perceived ageism with other important aspects of health, such as how people perceive their own memory (Kornadt et al., 2021).

One relatively under‐explored, yet significant, outcome of perceived ageism is subjective memory. The term subjective memory is used to indicate how individuals interpret, feel, or think about their own memory—in other words, one's perceptions regarding memory performance (Mogle et al., 2017). It is important to investigate one's self‐evaluations of memory since negative evaluations are related to greater risks of cognitive decline, dementia, and stroke, and are associated with biomarkers of Alzheimer's disease (Corlier et al., 2020; Sajjad et al., 2015), indicating a pre‐clinical disease stage that precedes objective cognitive symptoms (Buckley et al., 2016). Hence, poor subjective memory has been proposed as a first sign of cognitive decline, even before objective cognitive impairment can be detected using standard neuropsychological evaluations or clinical assessments (Mendonça et al., 2016; Reisberg & Gauthier, 2008). Subjective memory has also been found to affect functional health (Mendonça et al., 2016) and to be related to prior traumatic brain injury (Gardner et al., 2017). Although subjective memory is associated with depressive symptoms (Mogle et al., 2020), it nonetheless uniquely predicts increased risks of cognitive decline and dementia even after controlling for depressive symptoms (Carlos et al., 2020; Hülür et al., 2014; Jonker et al., 2000; Zullo et al., 2021). An investigation of Italian adults during the pandemic indicated a decline in subjective cognitive performance during lockdown compared to pre‐lockdown, while surprisingly finding improved subjective memory performance during lockdown (Fiorenzato et al., 2021). These mixed trends further highlight the need to investigate subjective memory trajectories during the pandemic. An advantage of measuring subjective memory in times of an infectious disease pandemic, when in‐person neuropsychological and clinical evaluations of cognitive status are limited, is that it is simple and quick to assess remotely and can be used to track the pandemic's effects on the health of older adults. Furthermore, we used a single item, taken from the US Health and Retirement Survey (HRS) to assess subjective memory, as is often done in similar studies (Abdulrab & Heun, 2008; Kuiper et al., 2017; Mitchell, 2008; Reid & MacLullich, 2006). The use of a single item allowed us to measure this construct effectively within the scope of a larger survey.

Increased societal ageism during the pandemic may translate into worsening subjective memory through a process of stereotype internalization, as proposed by the stereotype embodiment theory (Levy, 2009). The theory claims that, over the life course, people are exposed to a myriad of negative stereotypes about old age. These stereotypes are internalized and as people grow older, they come to embody these stereotypes (Levy, 2009). Therefore, negative societal perceptions of older adults can impact behavior by becoming a “self‐fulfilling prophecy” in later life and encouraging actions that are in line with one's perceptions of aging adults (Wurm et al., 2013). A similar concept is of age‐related self‐stigma, describing the tendency of older people to adopt negative definitions of old age. Accordingly, adults with more negative age‐related self‐stigma also have worse physical and mental health (González‐Domínguez et al., 2018). One of the most prevalent stereotypes of older adults is of them being incompetent and suffering from cognitive impairment, forgetfulness, and senility (Cuddy et al., 2005; Ory et al., 2003). Consequently, increased stigma may result in worse perceptions of one's own memory and, subsequently, more pronounced cognitive decline, thereby “fulfilling” this stereotype of old age (Levy, 2003).

The pandemic has brought the vulnerability of older adults to the forefront of policy and public discourse, thereby potentially making them even more susceptible to ageist stereotypes (Eibach et al., 2010). Physical distancing guidelines have further contributed to the social isolation and sedentary behaviors of older adults, potentially leading to worsening memory performance and its perception (Cohn‐Schwartz et al., 2022; Feter et al., 2021; Takechi et al., 2020). Evidence providing some support for this claim is also found outside of the pandemic context. Haslam et al. (2012) showed, for example, that adults who self‐categorized themselves as older and expected their own memory to decline in old age performed worse on memory tests. A different study found that older adults with fewer benevolent ageist experiences had better evaluations of their memory abilities via self‐compassion (Sublett & Bisconti, 2020). However, less is known about the effects of perceived societal ageism in relation to subjective memory, especially during the COVID‐19 pandemic. Since ageism during the COVID‐19 pandemic is a relatively new phenomenon, we developed a single item scale to assess perceptions of how older adults were treated by society as a whole during the pandemic (e.g., Cohn‐Schwartz & Ayalon, 2021). A single item allowed to assess this construct within the framework of a large longitudinal survey, while decreasing participant burden and facilitating the maintenance of a longitudinal sample.

To sum, this study aimed to examine the associations between perceptions of societal ageism during the COVID‐19 pandemic onset with changes in subjective memory among adults aged ≥55 year over a 10‐months period from April 2020 to January 2021 in the United States. We utilized a unique longitudinal survey that allowed fine‐grained tracking of subjective memory on a monthly basis as the pandemic progressed. We hypothesized that: 1) adults who perceived societal ageism to be higher at baseline (during the pandemic onset in spring 2020) would also report worse subjective memory at baseline, and 2) adults who perceived societal ageism to be higher at baseline would also report worsening subjective memory over time.

## METHODS

### Data and participants

Data were from the COVID‐19 Coping Study, a longitudinal mixed‐methods study of middle‐aged and older adults’ mental health and well‐being during the COVID‐19 pandemic in the United States (Kobayashi et al., 2021). Eligible participants were adults aged ≥55 years who resided in the United States, including Puerto Rico, and who were able to access and complete the online survey in English. Participants were recruited through an online, multi‐frame sampling strategy; full details on study design and recruitment are published elsewhere (Kobayashi et al., 2021). The study was conducted online to ensure it was in line with institutional limitations on in‐person research due to the COVID‐19 pandemic, and to reach as many people as possible who had Internet access. The current study utilized data from nine waves of measurement spanning 10 months, from April 2, 2020 to January 4, 2021. Data were collected once a month, with the baseline survey designed to take approximately 17 minutes and the subsequent monthly follow‐up surveys designed to take approximately 10 minutes. A total of 4453 participants were recruited into the longitudinal sample, of which 4444 had valid subjective memory data at baseline and they comprise our analytic sample. A total of 1340 participants provided valid data at all monthly follow‐ups, with participants completing an average of 5.5 measurement time points of subjective memory data for a total of 27,148 observations.

### Measures

*Subjective memory* was assessed at the baseline and each monthly follow‐up by asking participants, “How would you rate your memory at the present time?” with response options of “Poor” (0), “Fair” (1), “Good” (2), “Very Good” (3), and “Excellent” (4). Subjective memory is often assessed with a single question in the literature (Abdulrab & Heun, 2008; Kuiper et al., 2017; Mitchell, 2008; Reid & MacLullich, 2006). The item used in the current study was taken from the US Health and Retirement Survey (HRS), a well‐established and nationally representative longitudinal survey of US adults aged >50 years (Willis et al., 2006). Numerous studies used this measure from the US HRS have and found it to be associated with objective memory performance, traumatic brain injury and depressive symptoms (Gardner et al., 2017; Ha & Pai, 2018; Hülür et al., 2014; Mogle et al., 2020).

*Perceptions of societal ageism* were assessed at baseline by asking participants the extent to which they agreed with the statement: “The level of respect for older adults in society has decreased during the coronavirus pandemic,” with continuous response options of “Strongly disagree” (0), “Disagree” (1), “Neither agree nor disagree” (2), “Agree” (3), and “Strongly agree” (4). Since, ageism during the COVID‐19 pandemic is a relatively new phenomenon, there were no existing survey scales to evaluate this construct during the survey development. We thus developed this scale in collaboration with a survey methodologist at the Survey Research Center at the University of Michigan Institute for Social Research, using a commonly implemented 5‐point Likert‐style response scale, and pilot‐tested the item along with the full baseline survey prior to data collection (Kobayashi et al., 2021). We elected not to use existing ageism scales, as they typically assess interpersonal experiences of age‐based discrimination (Ayalon et al., 2019), while we aimed to examine perceptions of how older adults were treated by society as a whole during the pandemic. Since, the pandemic onset and since our scale was developed, there has been published work that used single‐item measures of perceived ageism during the pandemic (e.g., Cohn‐Schwartz & Ayalon, 2021).

*Covariates*: Socio‐demographic covariates that may be confounders of the relationship between perceived societal ageism and subjective memory were assessed at baseline: age (continuous), sex (male; female), education (less than a college or university degree; college or university degree or higher), race (white; non‐white), ethnicity (Hispanic or Latinx; Non‐Hispanic or Latinx), retirement status prior to the COVID‐19 pandemic (not retired; retired), living arrangement (living with others; living alone), number of physician‐diagnosed medical conditions (range: 0–7). The individual medical conditions asked about were high blood pressure, diabetes, heart disease, asthma, chronic obstructive pulmonary disorder, cancer, or another limiting, long‐standing condition. Other general health and mental health covariates were measured at baseline and each monthly follow‐up, and treated as time‐varying covariates: self‐rated health (poor; fair; good; very good; excellent), number of depressive symptoms in the past week, according to the eight‐item Center for Epidemiological Studies Depression (CES‐D) scale (Karim et al., 2015; range: 0–8), number of anxiety symptoms in the past week, according to the five‐item Beck Anxiety Inventory (Smith et al., 2017; range: 4–20), and loneliness score in the past week, according to the three‐item UCLA Loneliness Scale (Hughes et al., 2004; range 3–9).

### Statistical analysis

We described the baseline characteristics of the sample using means and percentages. We also examined the bivariate associations of the study variables at baseline with subjective memory at baseline, using t‐tests for dichotomous variables and Pearson correlations for continuous variables. We used longitudinal multilevel regression models (MLMs) to determine the rate of change in subjective memory across the measurement waves. MLM is a flexible approach for longitudinal analyses that accounts for repeated outcome measures (Rabe‐Hesketh & Skrondal, 2008). Power analysis conducted using the sjstats package (Lüdecke, 2019) suggests that a sample of at least 500 participants measured across nine time points would be adequate to identify an effect size (Cohen's d) of .10, with power of .80 and alpha of .05, indicating that our sample has adequate power. Missing data was handled by using MLMs, which use all available data points and are thus relatively robust to unbalanced data caused by study attrition or missing data. We performed data analysis using Stata version 16.1 (StataCorp, 2016). Monthly measurement wave was specified as the time metric, with the score of zero representing the baseline (reference) month of measurement. The analyses used the restricted maximum likelihood estimator (REML), and the models accounted for the random intercepts for respondents and random respondent slopes for linear and quadratic change. The initial study model estimated the trajectory of subjective memory over time with no covariates in the model, testing both a linear and a quadratic model to identify the overall trajectory. The second model examined the effects of perceived societal ageism on the intercept and slope of the subjective memory trajectory by adding an interaction of perceived societal ageism with measurement month. The third model added the baseline and time‐varying covariates, described above. We tested for statistical interactions between all covariates and the linear and quadratic rate of change; the final models retained all linear interactions and only those quadratic interactions that were statistically significant. The random effects suggested variability in the trajectories of subjective memory (random effects: between‐subject variance = .649, standard error [SE] = .016; within‐subject variance = .197, SE = .002). The intraclass correlation indicated that 77% of the total variance in subjective memory was due to between‐person variance.

## RESULTS

Characteristics of the sample are shown in Table 1. The mean age of the sample was 67 years (range: 55–99) and most participants were women, white, and with high education (Table 1). At baseline, participants reported an average of 2.14 depressive symptoms (out of 8), moderate levels of loneliness (mean score of 4.75 with a range of 3–9), and fairly good subjective memory—mean response of 2.82, corresponding to a mean response between “Good” [2] and “Very good” [3]. On average, participants responded with “Neither agree nor disagree” (2.04, range: 0–4) in response to the societal ageism claim, “The level of respect for older adults in society has decreased during the coronavirus pandemic.” We used that variable as continuous in the main analyses, but in order to better understand it we also examined how participants were divided in their responses to this claim: They were divided rather evenly, such that a total of 30% of participants agreed with this claim (responded “Agree” or “Strongly agree”), 33% disagreed (responded “Disagree” or “Strongly disagree”), and 37% reported neither agreeing nor disagreeing.

**TABLE 1:** Descriptive statistics at baseline and bivariate analyses with subjective memory at baseline, COVID‐19 Coping Study, United States, April 2020–January 2021, N = 4444

**Variable**	**Mean (SD)/N (%)**	**Range**	**Bivariate analyses with subjective memory** **(*r*/t‐tests)**
Subjective memory	2.82 (.94)	0–4	
Societal ageism	2.04 (1.04)	0–4	*r* = −.03
Age	67.22 (7.46)	55–99	*r* = −.01
Sex: Female	3188 (71.88%)		*t* = −3.76***
Education: High education	3572 (80.38%)		*t* = −9.89***
Race: White	4190 (94.28%)		*t* = −3.86***
Ethnicity: Hispanic or Latinx	128 (2.93%)		*t* = .27
Retired	2263 (50.95%)		*t* = −.26
Living alone	1167 (26.44%)		*t* = 2.46*
Chronic illnesses	1.18 (1.13)	0–7	*r* = −.19***
Self‐rated health	2.66 (.96)	0–4	*r* = .42***
Depressive symptoms	2.14 (2.29)	0–8	*r* = −.25***
Anxiety symptoms	8.47 (2.47)	4–20	*r* = −.18***
Loneliness	4.75 (1.70)	3–9	*r* = −.17***

*p < .05.

**p < .01.

***p < .001.

Table 1 also shows the bivariate associations between the covariates and subjective memory at baseline. Adults who perceived their memory as better were also more likely to be women, white, with high education, not living alone, had fewer chronic conditions, better self‐rated health, fewer depressive and anxiety symptoms, and less loneliness (Table 1). The correlation between baseline subjective memory and perceived societal ageism was marginally statistically significant (*p* = .052) and negative (*r* = −.03).

Table 2 shows results from the MLMs. The first model predicted overall subjective memory trajectories across the study period using linear and quadratic terms for time with no covariates in the model. Both the linear and quadratic slopes were statistically significant. The linear slope indicator was negative, Estimate (SE) = −.042 (.004), and the quadratic slope indicator was positive, Estimate (SE) = .003 (.001). These coefficients indicate that subjective memory followed a curve from April 2020 to January 2021, with a sharper decline in the initial months of the pandemic and a subsequent leveling off in rate of decline.

**TABLE 2:** Multilevel modeling results, COVID‐19 Coping Study, United States, April 2020–January 2021, N = 4444

	Model 1		Model 2		Model 3	
	Estimate	SE	Estimate	SE	Estimate	SE
Intercept	2.812***	(.014)	2.867***	(.029)	2.017***	(.150)
Month	−.042***	(.004)	−.029***	(.005)	−.105*	(.042)
Month X month	.003***	(.001)	.002***	(.000)	.013*	(.005)
Respect for adults decreased			−.027*	(.013)	.022	(.012)
Respect for adults decreased X month			−.006***	(.001)	−.005**	(.001)

*Note*: Model 1: effects of measurement month; Model 2: added agreement that respected for adults decreased during the COVID‐19 pandemic; Model 3 added covariates of age, sex, education, race, Hispanic/Latinx, retirement status, living alone, chronic illnesses, self‐rated health, depressive symptoms, anxiety symptoms, loneliness, and their interactions with measurement month.

*p < .05.

**p < .01.

***p < .001.

**FIGURE 1:**
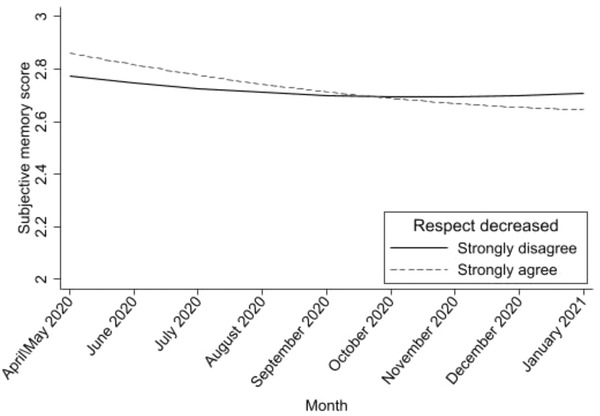
Estimated changes in subjective memory during the COVID‐19 pandemic according to perception of societal ageism, after controlling for the study covariates, COVID‐19 Coping Study, United States, April 2020–January 2021. The figure shows the estimated trajectories of subjective memory for the two extreme responses for the measure of perceived societal ageism (“Strongly disagree” and “Strongly agree”). Less extreme responses are not shown

The second model added perceived societal ageism at baseline (a continuous variable) as a predictor, showing a statistically significant negative association with subjective memory (Table 2; Model 2). As participants agreed to a greater extent that respect for older adults in society decreased during the pandemic, they were more likely to perceive their memory as worse at baseline, Estimate (SE) = −.027 (.013), *p* < .05. A higher perception of societal ageism was also related to a stronger linear decline in subjective memory, Estimate (SE) = −.006 (.001), *p* < .001 (Table 2). Even after the addition of sociodemographic, social, general health, and mental health covariates in the third model, adults who perceived that respect for older adults in society had declined during the pandemic experienced stronger declines in their subjective memory, Estimate (SE) = −.005 (.001), *p* < .01 (Table 2; Model 3, Figure 1). The association of perceived societal ageism with subjective memory at baseline became non‐significant. Figure 1 shows the estimated trajectories of subjective memory for the two extreme responses for the measure of perceived societal ageism (“Strongly disagree” and “Strongly agree”). Less‐extreme responses are not included to improve the clarity of the figure because they were intermediate to those shown on the figure.

## DISCUSSION

In this national longitudinal cohort study of US adults aged 55 and over, we observed that those who perceived society as more ageist at the COVID‐19 pandemic onset also experienced steeper declines in their subjective memory over the following 10 months. This finding was independent of covariates, including changes in mental health over time. This study is unique in its assessment of how adults perceived societal attitudes toward their age group during the early pandemic, thereby tapping into their subjective point of view in relation to society, and by evaluating the potential effects of these perceptions in relation to their subjective memory over a 10‐month period as the pandemic progressed. Given that subjective memory assessments are highly predictive of subsequent incidence of cognitive impairment, stroke, and dementia, future research should evaluate the longer‐term impact of perceived societal ageism on these important health outcomes.

This study offers novel insights into the factors contributing to decline in subjective memory during the pandemic, in accordance with the second study hypothesis. Partial support for the first hypothesis was seen in an association of societal ageism and worse subjective memory at baseline, but this became non‐significant following the addition of the covariates. Thus, it seems that the stronger effect is for change in subjective memory over time. Perceived societal ageism may affect subjective memory by being internalized and becoming a “self‐fulfilling prophecy” (Wurm et al., 2013). That is, older adults can internalize policies and media narratives that portray their age group as vulnerable and needing protection, narratives which were particularly prevalent during the early stages of the pandemic, for example in newspaper reporting (Jen et al., 2021). In accordance with the stereotype embodiment theory (Levy, 2009), these internalized stereotypes early on during the pandemic could make older adults believe they are indeed frail, a burden, and in need of assistance throughout later months and manifest in thinking of themselves as having worse memory. For example, they may notice more momentary lapses in memory, such as forgetting where they placed their keys, since these lapses match the negative stereotypes. They can then interpret such memory lapses as indicating poor overall memory. Another process through which ageism can be related to subjective memory is by causing stress via the societal focus on the vulnerability of older adults during the pandemic. Stress is known to affect late‐life cognition (Lupien et al., 2007) and adults who perceive society as becoming less respectful toward their age group could indeed experience worsening memory over time. This study adds to the literature on the health effects of perceived ageism, which often focuses on mental and physical health (Kornadt et al., 2021), by showing its impact in relation to subjective memory.

Understanding the wide‐reaching effects of the pandemic can facilitate targeting those adults who experience ageism and could thus be at a greater risk for longer‐term cognitive declines in the wake of the pandemic. Social policies can combat the ageist perceptions and policies that have proliferated during the pandemic (Monahan et al., 2020). Policymakers should emphasize the equality of older adults to younger age groups, avoid grouping all adults into a single homogenous group, and refrain from policies that marginalize or discriminate against them. We also need more concerted efforts to decrease societal age‐based segregation (Hagestad & Uhlenberg, 2005). Practitioners and community service providers working with older populations can encourage connections between people of different ages (Drury et al., 2022). Programs that provide aging education and facilitate positive intergenerational relationships with older adults could be especially fruitful (Jarrott et al., 2022; Lytle & Levy, 2019). An example of such a program is the “Instapals” program, which facilitated contact between undergraduate students and older adults through daily exchanges on Instagram and in‐person meetings. The program yielded positive results in relation to improving attitudes toward older adults (Lytle et al., 2020). Such online programs are also relevant to the pandemic, as they comply with COVID‐19 public health recommendations. As intergenerational contact is one of the most effective means of reducing ageism and self‐directed ageism (Burnes et al., 2019; Cohn‐Schwartz et al., 2022), such programs can promote the perception by older adults that they are a valued and respected part of society. We note, however, that policymakers should take into consideration the digital divide among older adults when designing programs for this population (Huxhold et al., 2020). Thus, any program based on online access and ability should include means to overcome the technical challenges that some older adults may face, in order to be truly inclusive and reach a wide array of older adults.

The present study also provided a unique longitudinal investigation of the population trajectory of subjective memory during the COVID‐19 pandemic and indicates that the subjective memory of middle‐aged and older adults' has somewhat declined, especially during the early months of the pandemic. While the decline in subjective memory is not extremely steep, there is a significant decline which could manifest in a worsening evaluation of one's memory and consequently in negative behavioral outcomes. Worsening subjective memory can have implications for cognitive function and mental health (Hülür et al., 2014; Jonker et al., 2000; Zullo et al., 2021), as well as be an early marker for cognitive decline and dementia (Mendonça et al., 2016; Reisberg & Gauthier, 2008). Since perceiving one's memory as worse can have negative implications for everyday life, including on quality of life and daily physical functioning, poor subjective memory could have provided adults with an additional source of stress in an already difficult and stressful period. Our statistical control for depressive and anxiety symptoms over time strengthens the claim that these results are not simply due to depression and anxiety experienced during the pandemic.

As subjective memory deteriorated and remained relatively low toward the beginning of 2021 in this study population, the results of this study may attest to relatively long‐lasting effects of the pandemic on the subjective memory of middle‐aged and older adults. Future investigations should follow‐up on the subjective memory of adults in later stages of the pandemic to assess whether these effects shift as the pandemic situation changes. Policymakers should strive to identify adults with subjective memory declines and work toward protecting older adults’ cognitive health in the wake of the pandemic. Such policy measures could include routine screening for memory declines when older adults come to medical centers and clinics, as well as implementing social programs to improve cognitive function (Wesselman et al., 2019).

This study has limitations. Some of the effects on subjective memory could stem from the COVID‐19 disease itself, as evidence indicates its harmful effects on cognitive functioning among older adults with or without cognitive impairment or dementia (Manca et al., 2020; Søraas et al., 2021). However, our sample had a low rate of self‐reported COVID‐19 diagnoses (e.g., <1% at baseline), limiting our ability to fully examine such effects. Additionally, the study was based on a convenience sample of adults who were able to complete online questionnaires in English, resulting in a sample of relatively highly educated and mostly white adults, potentially excluding those who may have been most affected by the pandemic, including racial/ethnic minority older adults who are at higher risk for COVID‐19 infection and death (Millett et al., 2020). If those who were not represented in this study experienced greater perceived societal ageism and worse subjective memory than those who took part, our estimates may be conservative. Although the online study recruitment and data collection methods allowed us to rapidly collect rich data as the pandemic evolved and in‐person research interviews were impossible, future studies should represent more diverse samples. In addition, we used single measures of subjective memory and perceived ageism. The use of such brief measures reduced participant burden and helped us to maintain a longitudinal sample, and these measures have been used successfully in the literature (Gardner et al., 2017; Ha & Pai, 2018; Hülür et al., 2014; Mogle et al., 2020). Future research should use more extensive measures to examine more fine‐grained and comprehensive aspects of these constructs, for example by looking at different aspects of ageism and not only ageism in general (e.g., Kornadt et al., 2021). Our measure of societal ageism during the COVID‐19 pandemic was newly developed for the purpose of this study, and while it uses a standard 5‐point Likert‐style format, it should be assessed for validity and reliability in future work. Longitudinal measures of societal perceptions of ageism are needed to elucidate time‐varying associations between ageism and memory.

In conclusion, this timely study identified longitudinal deteriorations in subjective memory in this sample of middle‐aged and older US adults, which were especially pronounced in the initial months of the COVID‐19 pandemic. This trend was more strongly observed among adults who believed respect for older adults decreased during the pandemic. Understanding the effects of the pandemic on middle‐aged and older adults' subjective memory can facilitate targeting those who may be at elevated risk for cognitive decline and dementia in the wake of the pandemic. These results imply that societal interventions should combat ageism that has proliferated during this period, as this study indicates it may have negative implications for subjective memory among middle‐aged and older adults.
